# Supplementary material for: Insulin at the intersection of thermoregulation and glucose homeostasis
Source: Mol Metab. 2024 Feb 13;81:101901. doi: 10.1016/j.molmet.2024.101901 (PMC10877958; doi:10.1016/j.molmet.2024.101901)
Supplement: Multimedia component 1 [file mmc1.docx]

**Supplemental Material**

**Insulin at the Intersection of Thermoregulation and Glucose Homeostasis**

Nathan C. Winn^1*^, Michael W. Schleh^1^, Jamie N. Garcia^1^, Louise Lantier^1,2^, Owen P. McGuinness^1,2^, Joslin A. Blair^1^, Alyssa H. Hasty^1,3^, David H. Wasserman^1,2^

^1^Department of Molecular Physiology and Biophysics, Vanderbilt University, Nashville, Tennessee, USA

^2^Vanderbilt Mouse Metabolic Phenotyping Center, Nashville, Tennessee, USA

^3^VA Tennessee Valley Healthcare System, Nashville, Tennessee, USA

**
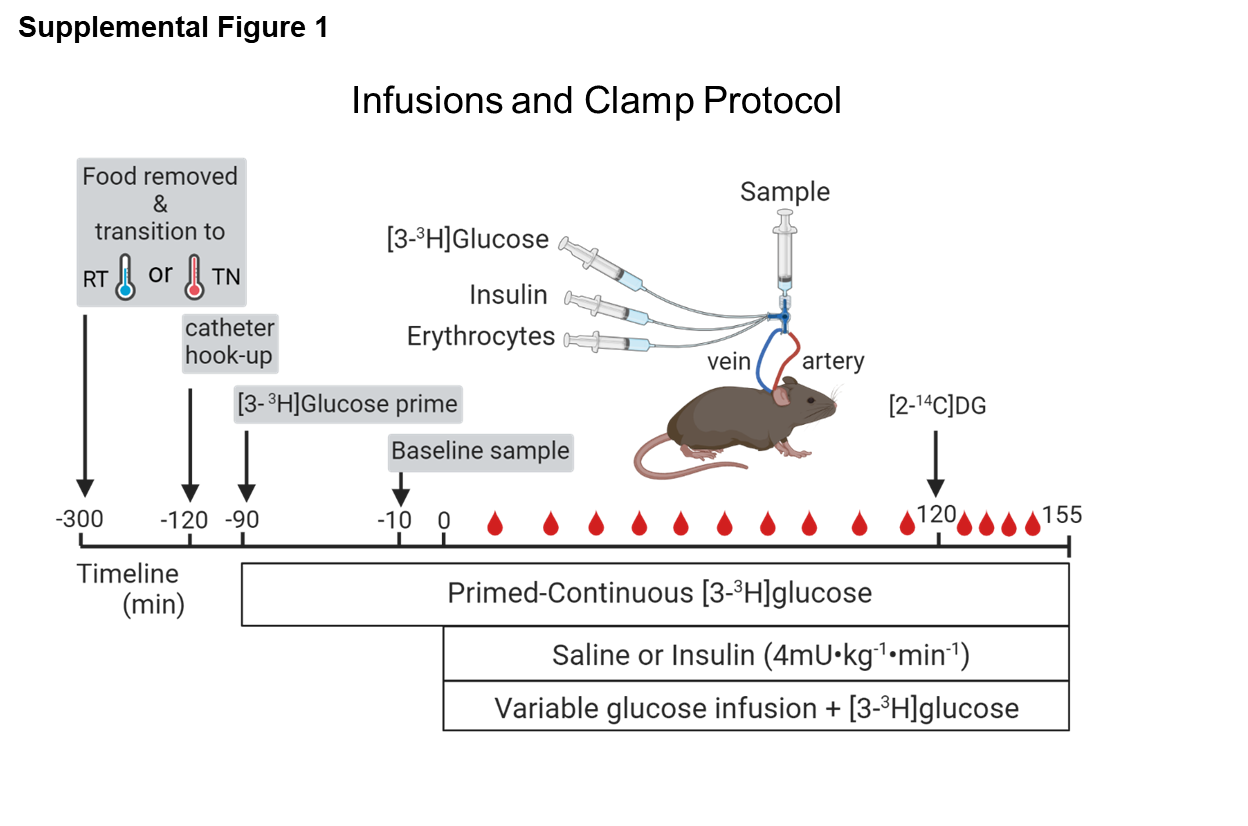
**

**Supplemental Figure 1:** Experimental vehicle and insulin clamp schematic description.

**Supplemental Table 1**

| **Ratio of Tissue [^14^C]2DG/Arterial [^14^C]2DG** | | | | | | | |
| --- | --- | --- | --- | --- | --- | --- | --- |
|  | **Vehicle** | | **Insulin** | | ANOVA | | |
| Tissue | **RT→RT** | **RT→TN** | **RT→RT** | **RT→TN** | Temp | Insulin | Interaction |
| BAT | 1.39 ± 0.26 | $0.33 ± 0.07 | 1.47 ± 0.24 | #0.90 ± 0.22 | **p=0.0009** | p=0.14 | p=0.26 |
| Heart | 1.20 ± 0.10 | 1.10 ± 0.14 | 1.61 ± 0.11 | 1.58 ± 0.15 | p=0.65 | **p=0.002** | p=0.77 |
| Gastroc | 0.17 ± 0.01 | 0.12 ± 0.01 | 0.24 ± 0.02 | #0.34 ± 0.05 | p=0.40 | **p<0.0001** | **p=0.01** |
| Vastus L. | 0.16 ± 0.01 | 0.13 ± 0.01 | 0.25 ± 0.02 | 0.30 ± 0.05 | p=0.71 | **p=0.0006** | p=0.32 |
| eWAT | 0.21 ± 0.04 | 0.17 ± 0.01 | 0.16 ± 0.02 | 0.35 ± 0.09 | p=0.20 | p=0.26 | p=0.06 |
| iWAT | 0.36 ± 0.04 | 0.39 ± 0.02 | 0.32 ± 0.03 | 0.63 ± 0.18 | p=0.12 | p=0.35 | p=0.20 |
| The arterial [^14^C]2DG measurement was made at t=145 min. Tissues were simultaneously excised and snap frozen for measurement of unphosphorylated [^14^C]2DG. Unphosphorylated [^14^C]2DG is assumed to be constrained to the extracellular space. Tissue radioactivity was measured and normalized per mg tissue weight. Radioactivity in dpm/mg were converted to dpm/mL tissue using empirical ratios of total tissue water (TW) and extracellular water (ECW): BAT (TW=0.50, ECW=0.50) (1); Heart (TW=0.75, ECW=0.25) (2); Skeletal muscle (TW=0.75, ECW=0.20) (3); and WAT (TW=0.14, ECW=0.75) (3, 4). Two-way ANOVA with Temperature and Insulin as factors was run to detect differences for main effects and the interaction term. n=5-9/group. Data are mean ± SE. dpm, disintegrations per minute.  $p<0.05 vs RT→RT vehicle  #p<0.05 vs RT→RT insulin | | | | | | | |

In both temperature conditions, insulin increases this ratio in the heart and skeletal muscle, but not adipose tissue (**Supplemental Table 1**). Compared to RT→RT, RT→TN reduces the tissue [^14^C]2DG to arterial ratio in BAT; whereas it is increased in the gastrocnemius muscle. Interestingly, the ratio in heart is >1.0, as it is in insulin-stimulated BAT during RT→RT. A ratio of tissue to arterial [^14^C]2DG >1.0 indicates the presence of intracellular [^14^C]2DG. This results when phosphorylation of [^14^C]2DG is inadequate to match the intracellular transport of [^14^C]2DG (5).

**Supplemental Table 2**

| **Ratio of Tissue [^14^C]2DG/Arterial [^14^C]2DG** | | | | | | | |
| --- | --- | --- | --- | --- | --- | --- | --- |
|  | **Vehicle** | | **Insulin** | | ANOVA | | |
| Tissue | **TN→TN** | **TN→RT** | **TN→TN** | **TN→RT** | Temp | Insulin | Interaction |
| BAT | 0.27 ± 0.04 | 0.58 ± 0.18 | 0.70 ± 0.17 | #1.35 ± 0.23 | **p=0.02** | **p=0.002** | p=0.29 |
| Heart | 0.38 ± 0.04 | 0.43 ± 0.08 | 1.45 ± 0.11 | #1.86 ± 0.18 | p=0.13 | **p<0.0001** | p=0.23 |
| Gastroc | 0.12 ± 0.01 | 0.13 ± 0.01 | 0.32 ± 0.04 | 0.32 ± 0.02 | p=0.96 | **p<0.0001** | p=0.84 |
| Vastus L. | 0.12 ± 0.01 | 0.14 ± 0.02 | 0.30 ± 0.05 | 0.31 ± 0.02 | p=0.75 | **p<0.0001** | p=0.87 |
| eWAT | 0.08 ± 0.01 | 0.10 ± 0.01 | 0.18 ± 0.03 | 0.20 ± 0.04 | p=0.52 | **p=0.005** | p=0.98 |
| iWAT | 0.23 ± 0.03 | 0.23 ± 0.01 | 0.31 ± 0.05 | #0.46 ± 0.04 | p=0.057 | **p=0.0007** | p=0.07 |
| The arterial [^14^C]2DG measurement was made at t=145 min. Tissues were simultaneously excised and snap frozen for measurement of unphosphorylated [^14^C]2DG. Unphosphorylated [^14^C]2DG is assumed to be constrained to the extracellular space. Tissue radioactivity was measured and normalized per mg tissue weight. Radioactivity in dpm/mg were converted to dpm/mL tissue using empirical ratios of total tissue water (TW) and extracellular water (ECW): BAT (TW=0.50, ECW=0.50) (1); Heart (TW=0.75, ECW=0.25) (2); Skeletal muscle (TW=0.75, ECW=0.20) (3); and WAT (TW=0.14, ECW=0.75) (3, 4). Two-way ANOVA with Temperature and Insulin as factors was run to detect differences for main effects and the interaction term. n=5-9/group. Data are mean ± SE. dpm, disintegrations per minute.  #p<0.05 vs TN→TN | | | | | | | |

In TN-adapted groups, insulin increases the ratio of tissue [^14^C]2DG/arterial [^14^C]2DG in all tissues measured (**Supplemental Table 2**). Compared to TN→TN, TN→RT increases the ratio in BAT and iWAT during the insulin clamp. Similar to RT-adapted mice, the ratio in the heart is >1.0 and >1.0 in insulin-stimulated BAT in TN→RT.

**
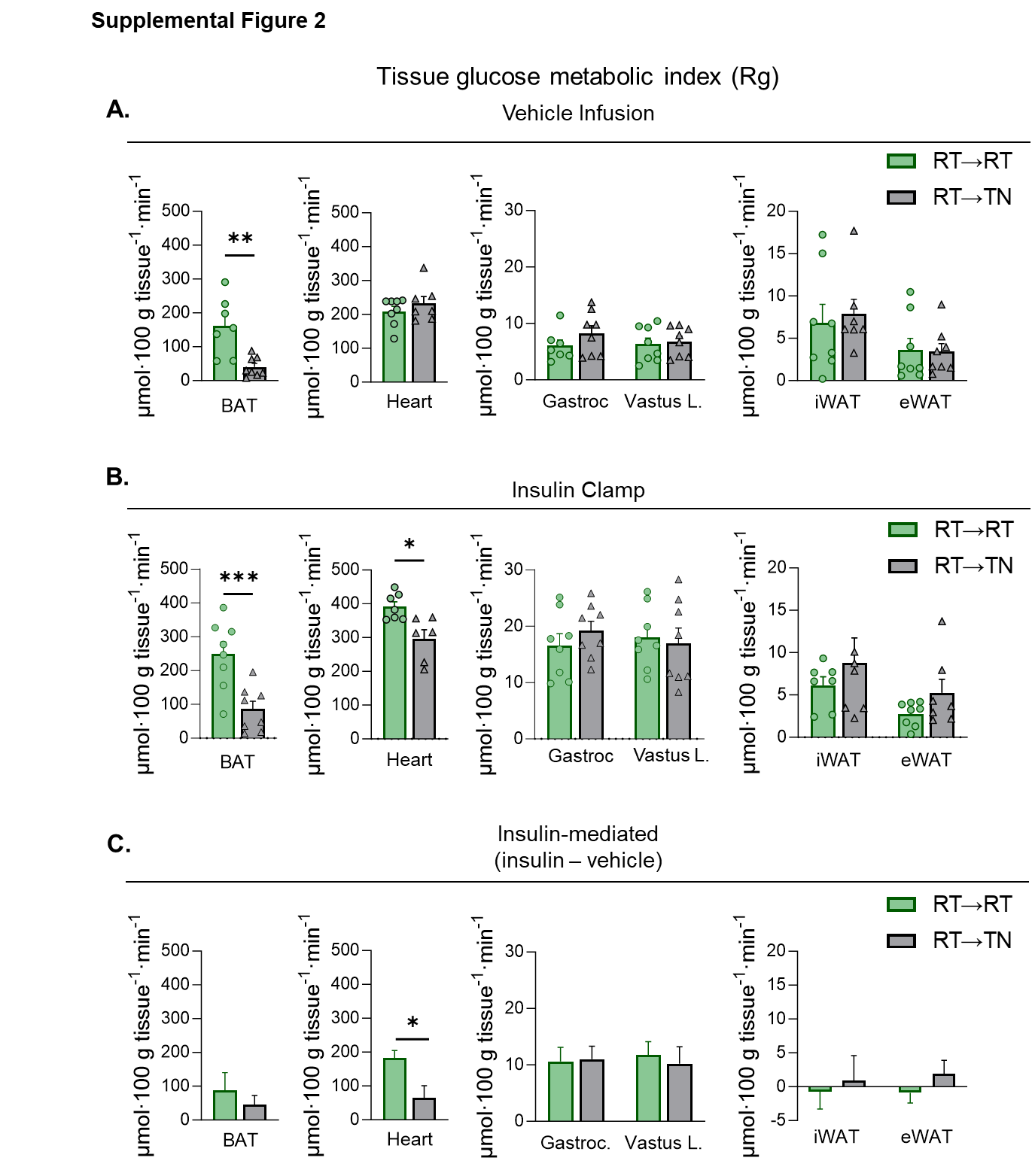
**

**Supplemental Figure 2 *- Non-insulin and insulin-mediated tissue glucose metabolic index during short-term transition from RT-adapted to TN (RT→TN)*.**  [^14^C]2-deoxyglucose was infused as a bolus at t=120 of each respective clamp. Blood was collected frequently for 25 minutes to determine the rate of disappearance using exponential decay. Tissues were rapidly excised and snap frozen for isotopic enrichment. Tissue Rg between RT→RT and RT →TN during **A**) vehicle and **B**) insulin clamps. **C**) Insulin-stimulated tissue Rg was computed as the mean differences in tissue Rg between insulin and vehicle infusions. The variance between vehicle and insulin clamps were calculated using the standard error of the difference. Statistical significance was determined using a t-distribution table with critical t-values and corresponding degrees of freedom. Data are mean ± SE; n=6-9/group. p<0.05 was used to reject the null hypothesis. *p<0.05, **p<0.01, ***p<0.001

**
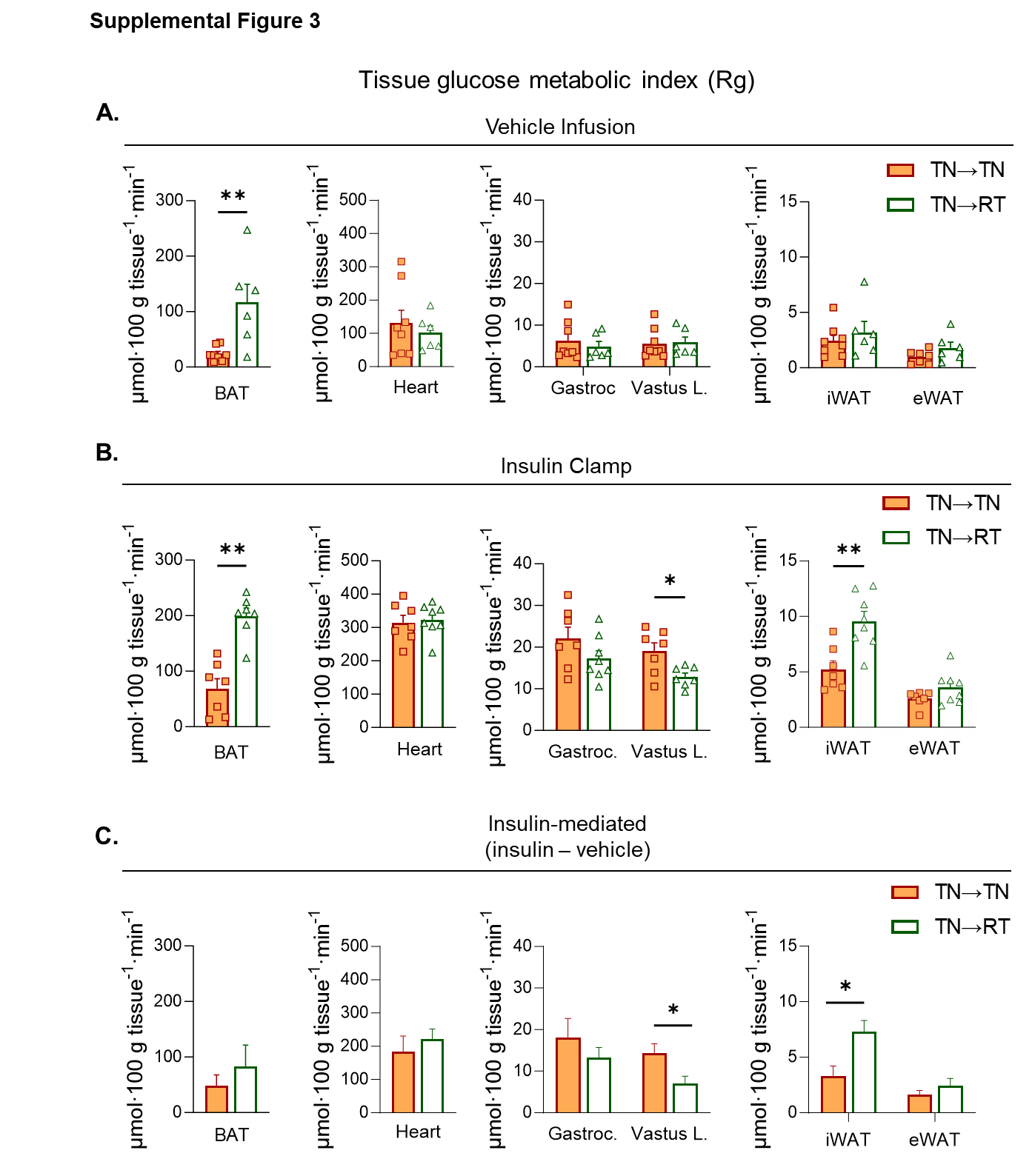
**

**Supplemental Figure 3** - ***Non-insulin and insulin-mediated tissue glucose metabolic index during short-term transition from TN-adapted to RT (TN→RT)***. [^14^C]2-deoxyglucose was infused as a bolus at t=120 of each respective clamp. Blood was collected frequently for 25 minutes to determine the rate of disappearance using exponential decay. Tissues were rapidly excised and snap frozen for isotopic enrichment. Tissue Rg between TN→TN and TN →RT during **A**) vehicle and **B**) insulin clamps. **C**) Insulin-stimulated tissue Rg was computed as the mean differences in tissue Rg between insulin and vehicle infusion. The variance between vehicle and insulin clamps were calculated using the standard error of the difference. Statistical significance was determined using a t-distribution table with critical t-values and corresponding degrees of freedom. Data are mean ± SE; n=6-9/group. p<0.05 was used to reject the null hypothesis. *p<0.05, **p<0.01

**Supplemental Figure 4**

**
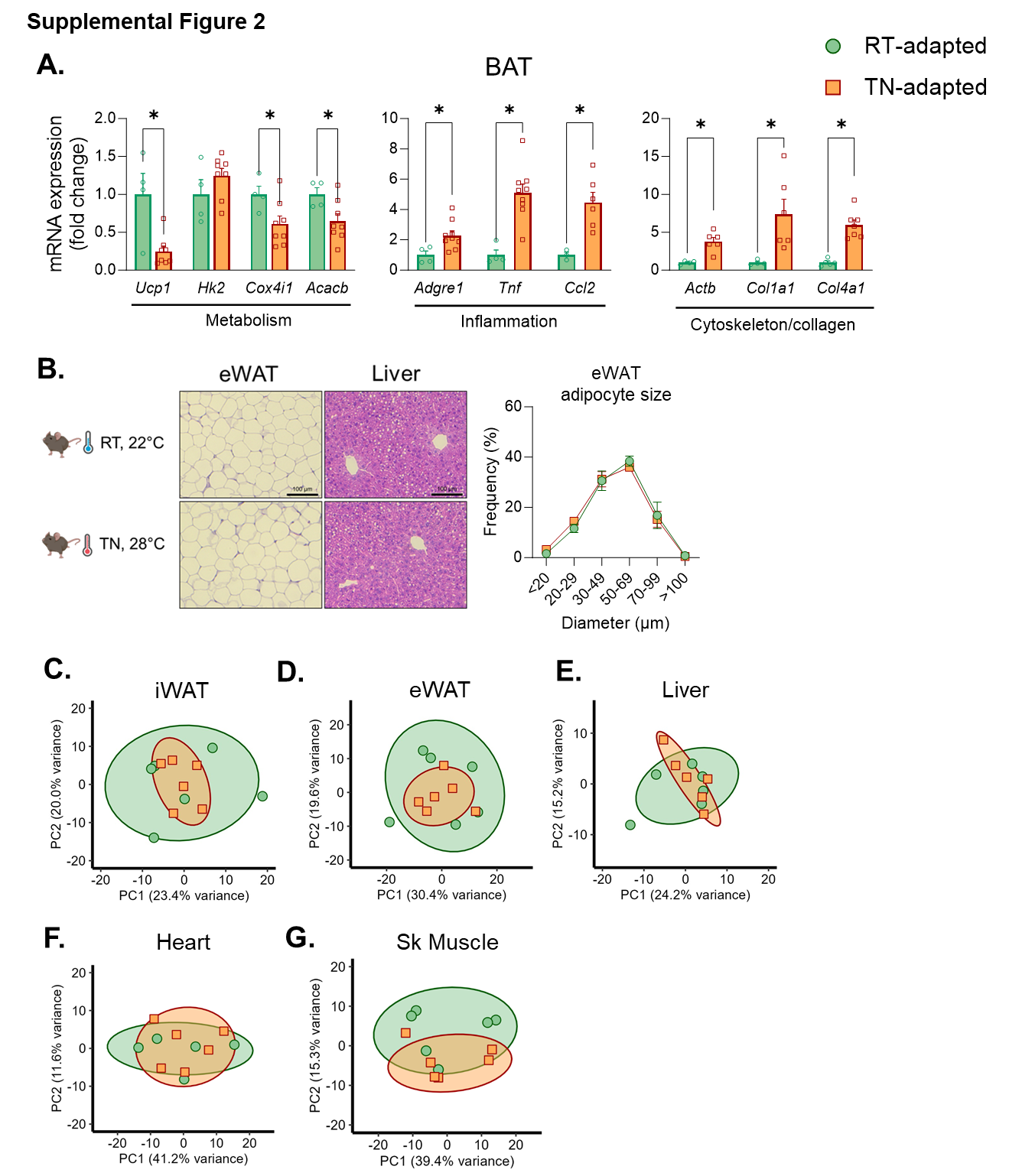
**

**Supplemental Figure 4:** Mice were adapted to RT or TN for 6 weeks. **A**) mRNA expression in brown adipose tissue (BAT) from TN-adapted vs RT-adapted mice. **B**) eWAT and liver micropgraphs were generated. Adipocyte size was calculated via ImageJ and presented as frequency per bin size. After a 5 h fast, plasma, liver, heart, gastrocnemius muscle, inguinal adipose tissue (iWAT), and epididymal adipose tissue (eWAT) were quickly excised and flash frozen in liquid nitrogen from mice acclimated to RT or TN. Tissues were processed and analyzed using HILIC negative ion mode mass spectrometry (LC MS). Spectral intensities were median normalized and log10 transformed. PCA plots were generated via R software package in **C**) iWAT, **D**) eWAT, **E**) liver, **F**) heart, and **G**) skeletal muscle (Sk muscle). n=4-8/group. Student t tests were run to determine statistically significant difference. *p<0.05

**
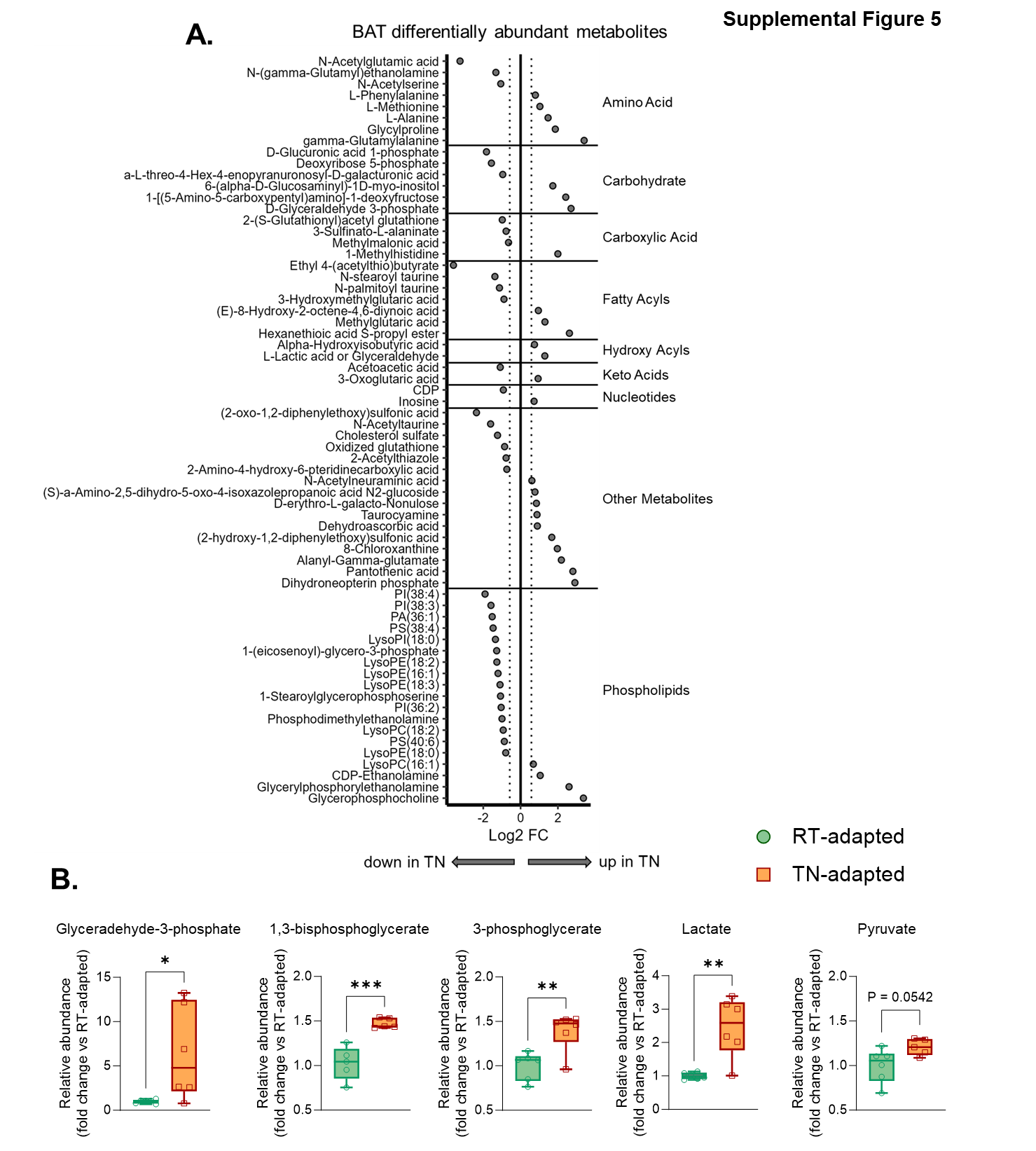
Supplemental Figure 5**: Mice were adapted to RT or TN for 6 weeks. A) Differentially expressed BAT metabolites are presented relative to RT-adapted control. Metabolites are clustered by class and presented as Log2 fold difference. B) Relative differences in glycolytic intermediates are presented as fold change from RT group. Significance was accepted if FDR<0.05.

**
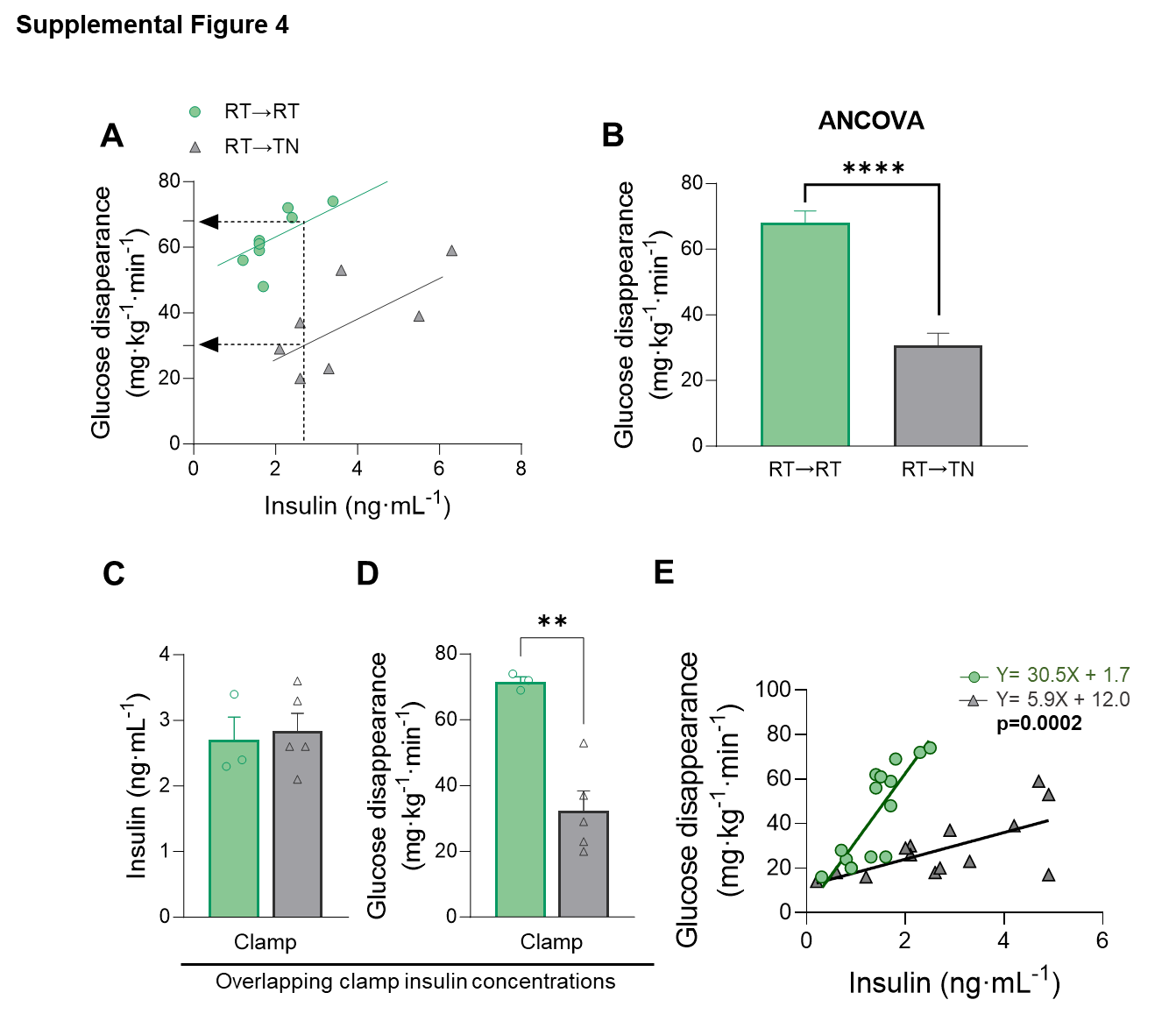
Supplemental Figure 6**

**Supplemental Figure 6 – A**) The ANCOVA model fit depicting a single pooled slope estimate for the two groups. The arrows point to the adjusted group mean glucose disappearance values at a given insulin concentration. The difference in elevations is the adjusted group difference in glucose disappearance. **B**) ANCOVA adjusted glucose Rd with clamp arterial insulin entered into the model as ‘grand mean’ (grand mean is the average clamp insulin level for combined RT→RT and RT→TN). **C**) Arterial insulin and **D**) glucose Rd in a subset of mice with matched ‘clamp’ insulin concentrations. After matching individual insulin levels between groups, glucose Rd remained lower in RT→TN mice during the insulin clamp. **E**) The relationship between glucose Rd and insulin from basal (no insulin infusion) to steady clamp conditions. In this representation, a shift of the curve to the right with a flattened slope is indicative of decreased insulin action. Data are mean ± SE. n=3-5/group. **p<0.01, ****p<0.001

**
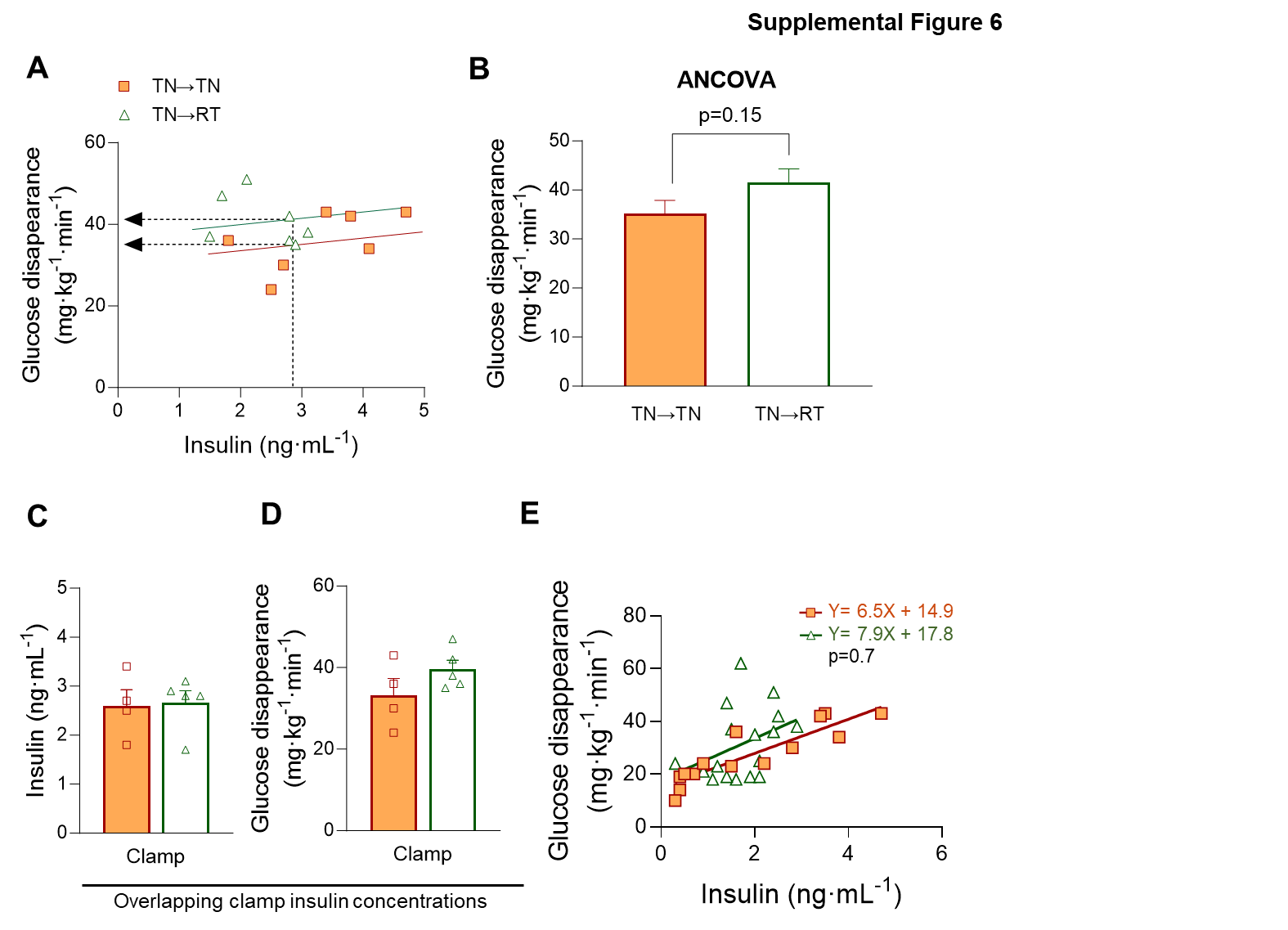
Supplemental Figure 7**

**Supplemental Figure 7:** – **A**) The ANCOVA model fit depicting a single pooled slope estimate for the two groups. The arrows point to the adjusted group mean glucose disappearance values at a given insulin concentration. The difference in elevations is the adjusted group difference in glucose disappearance. **B**) ANCOVA adjusted glucose disappearance with clamp arterial insulin entered into the model as ‘grand mean’ (grand mean is the average clamp insulin level for combined RT→RT and RT→TN). **C**) Arterial insulin and **D**) glucose disappearance in a subset of mice with matched ‘clamp’ insulin concentrations. After individual matching of clamp insulin levels between groups, glucose disappearance was not significantly different between TN→TN and TN→RT mice. **E**) The relationship between glucose Rd and insulin from basal (no insulin infusion) to steady clamp conditions. In this representation, a shift of the curve to the right with a flattened slope is indicative of decreased insulin action. Data are mean ± SE. n=4-5/group.


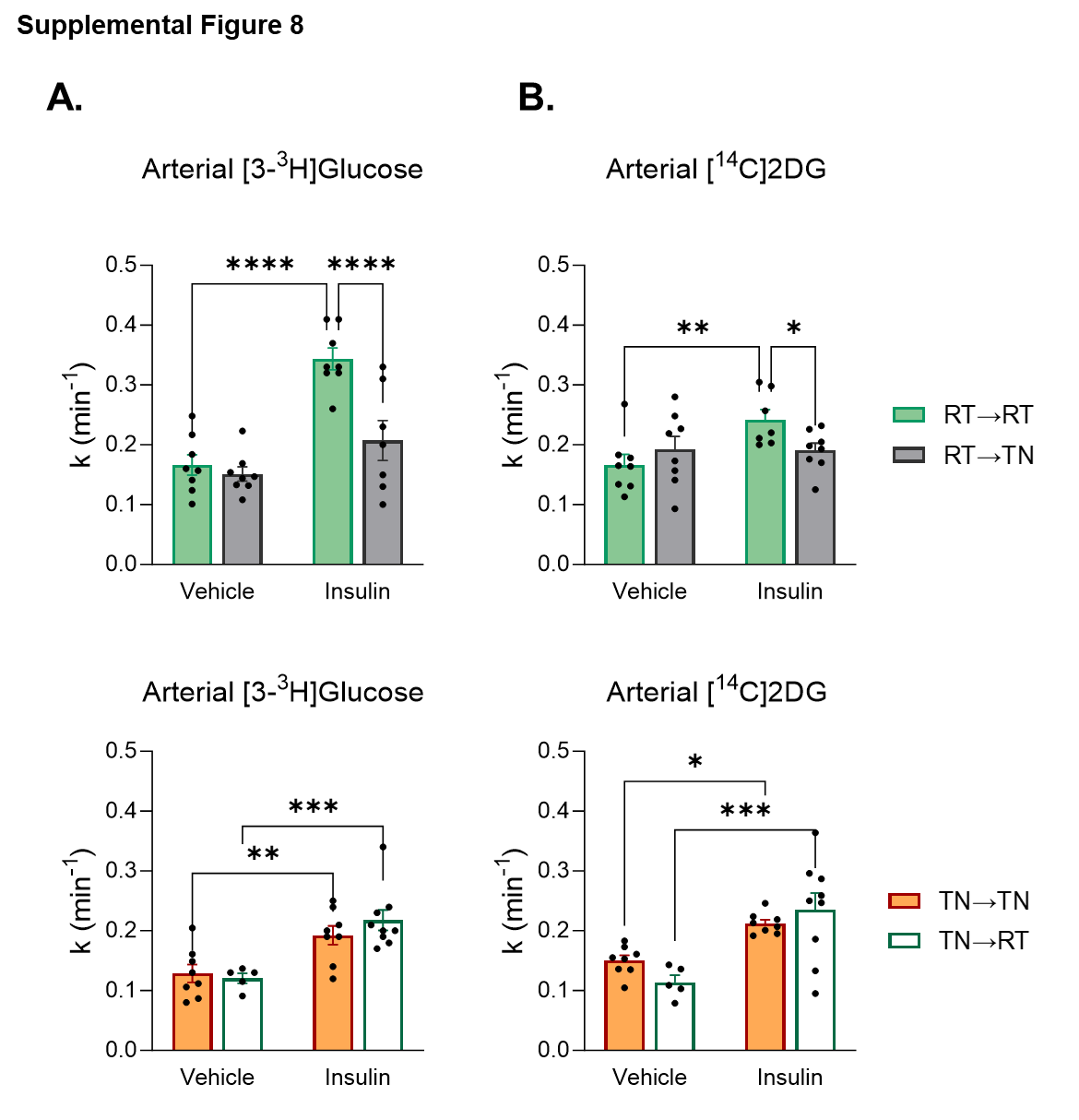


**Supplemental Figure 8** - Rate constants from A) arterial [3-^3^H]glucose and B) arterial [^14^C]2DG measurements in RT→TN versus RT→RT and TN→TN versus TN→RT during vehicle infusion or insulin clamp. Two way ANOVA with Temperature (RT and TN) and treatment (vehicle and insulin) as factors were run. Data are mean ± SE. n=5-9/group.

**REFERENCES**

1. Branca RT, Zhang L, Warren WS, Auerbach E, Khanna A, Degan S, et al. In vivo noninvasive detection of Brown Adipose Tissue through intermolecular zero-quantum MRI. *PLoS One.* 2013;8(9):e74206.

2. Andres-Villarreal M, Barba I, Poncelas M, Inserte J, Rodriguez-Palomares J, Pineda V, et al. Measuring Water Distribution in the Heart: Preventing Edema Reduces Ischemia-Reperfusion Injury. *Journal of the American Heart Association.* 2016;5(12).

3. Marken Lichtenbelt WD, and Fogelholm M. Increased extracellular water compartment, relative to intracellular water compartment, after weight reduction. *J Appl Physiol (1985).* 1999;87(1):294-8.

4. Pierson RN, Jr., wang J, Yang MU, Hashim SA, and Van Itallie TB. The assessment of human body composition during weight reduction: evaluation of a new model for clinical studies. *J Nutr.* 1976;106(12):1694-701.

5. Russell RR, 3rd, Mrus JM, Mommessin JI, and Taegtmeyer H. Compartmentation of hexokinase in rat heart. A critical factor for tracer kinetic analysis of myocardial glucose metabolism. *J Clin Invest.* 1992;90(5):1972-7.
